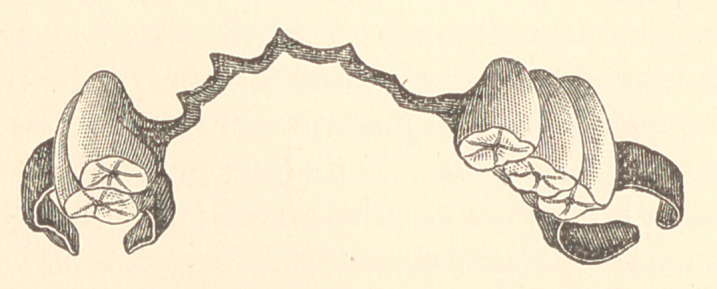# Crown- and Bridge-Work

**Published:** 1892-10

**Authors:** C. M. Richmond

**Affiliations:** New York


### CROWN- AND BRIDGE-WORK.[1]

BY DR. C. M. RICHMOND, NEW YORK.

(Continued from page 637.)

In my article for this month I give an upper partial case, in which both bicuspids on one side and both bicuspids and one molar on the other are missing.

The patient had worn a gold plate, and came to have it replaced with bridge-work. The teeth remaining all good, I decided to put in a movable case and not cut any of the teeth in the slightest degree. This pleased the patient, as she was extremely sensitive and had been through enough before, and to have a difficult case made and no pain and worry was half the battle.

I first took a plaster impression of the two molars I wished to use in holding the case in position, and cast fusible metal dies. Bands of gold were then made to fit the dies as nearly as possible without the use of a hammer; they were then soldered together at the point where they are to be cut apart after the next band of clasp-metal is soldered on to them.

These bands were made the same as those I have described in a

---

[1] Copyright, 1892, by Dr. C. M. Richmond.

former article, by soldering a clasp band on to a gold band which had been previously fitted perfectly to the die of fusible metal, the front or wire support being made of hard wire, or what is termed irido-platinum No. 16. An impression is taken of the six front teeth in plaster, and a cast of a metal die is also made; wire is now cut and bent to fit the shape of each tooth, letting the ends meet at the space at the necks; so it will be seen that this wire part is made of six pieces. After each piece is so fitted as to make a perfect joint, they are waxed together where they meet with a hot spatula and hard wax. This is done on the die; the wire is then carefully removed and invested, leaving the part exposed where the wax is. After the wax is removed the investment is put into the fire and brought to the proper temperature, and each piece of wire is soldered to the other piece, always with pure gold. I use pure gold for the reason that when heat enough has been applied to melt pure gold the wire will be securely soldered, and when solder is used it is not so perfect as pure gold soldering, and will not be strong, while the other is as strong as a whole wire.

The wire and clasps being finished, the clasps are placed in position on the teeth, and a plaster impression is taken, being careful to get the space where the teeth are lost, also the front teeth at the gum-line, perfect. The clasps are now removed from the teeth and placed in the impression. The bands are filled with investment and the balance of the cast is made of plaster; this is all poured at one time. (The reason is, that when I have the teeth placed in position, and the bands and wire and teeth are all waxed together, with a sharp knife I can cut the bands loose from the model and still have them invested.)

After the impression has been cut away we have a model which is a fac-simile of the mouth, with the bands in position. The next step is to place a piece of pure gold, 30 thick, on the model, directly above the position the teeth are to occupy, and by a little care this can be easily and perfectly fitted to the plaster cast by burnishing. After this has been done I place the wire front in position on the model, and the work is then completed up to the fitting of the teeth. The four bicuspids and one molar are selected the right length and width to fill the spaces, allowing enough for grinding the teeth to fit the gum perfectly. Plain plate teeth are used, and, after grinding, they are backed up with pure gold and waxed into position. We now have the bands, the small gold plate on the gum-line directly under the teeth, the wire front, and the teeth all waxed together on the model in the position it is to occupy when finished. We are now ready for the last investment, and by taking a sharp knife and carefully cutting the molar teeth (which hold the bands) off at the neck, the case can be taken off of the cast, and it has the same appearance that it has in the finished case, excepting that the bands are filled with investment. It is now invested, the solder is melted into position with the blow-pipe until the space between the teeth and the gold plate is filled, the ends of the wire front are at the same time soldered into the case, and simultaneously the bands are fastened to the work.

This case is worn by the patient, who says she is absolutely unconscious of its presence in her mouth, so perfect is the adjustment.

Should the bands get the least sprung they can be at once tightened so that the case will fit as well as when first made.

This class of operations, if they are perfectly made and properly adjusted, are the most satisfactory I have ever prepared, and the operation is applicable to almost all cases.

The case shows one very irregular tooth, and the other one is quite symmetrical and perfect. The bands are left open at the distal surface where the teeth join the next molars, but they could be left open at the point most advantageous to the work. I frequently leave them open, one on the inside and one on the outside, or both inside or both outside, as the case presents itself. Each case has its own requirements, and the operator must use his judgment and skill in mastering the different phases which are presented.

Should any one wish to get the fusible metal I use in these operations, it can be obtained from R. S. Williams, whom I have induced to keep it in stock.

(To be continued.)

## Figures and Tables

**Figure f1:**